# Meningioma preceding CASPR2 antibody limbic encephalitis with a stroke mimic: A case report

**DOI:** 10.1097/MD.0000000000026241

**Published:** 2021-06-11

**Authors:** Xiao-Jin Lu, Ran Li, Yong-Xing Chen, Xian-Kai Xu, Bao-Lin Shi

**Affiliations:** a Department of Neurology, School of Clinical, Weifang Medical University; b Department of Neurology, Affiliated Traditional Chinese Medicine Hospital of Weifang Medical University; c Department of Neurology, Weifang People's Hospital (The First Affiliated Hospital of Weifang Medical University), Weifang, Shandong, China.

**Keywords:** CASPR2 antibody, limbic encephalitis, meningioma, stroke mimic

## Abstract

**Rationale:**

Todd paralysis (a stroke-like presentation in some patients with epilepsy) caused by limbic encephalitis (LE) is not easily distinguished from acute ischemic stroke by clinicians in the emergency room.

**Patient concerns:**

We report a contactin-associated protein-like 2-antibody (CASPR2-Ab)-positive patient who presented with atypical LE.

**Diagnoses:**

CASPR2-Ab-positive LE was the presumed diagnosis. Re-evaluation of cerebrospinal fluid (CSF) samples revealed autoantibodies targeting CASPR2 at an immunoglobulin G titer of 1:1. The clinical presentation of subacute onset seizures, abnormal electroencephalography, hypermetabolism on positron emission tomography, good immunotherapy response, and the presence of specific antibodies in serum supports a diagnosis of autoimmune LE.

**Intervention:**

The patient received glucocorticoids (1 g for 3 days and 500 mg for 3 days), immunoglobulin (25 g for 3 days), sodium valproate (1 g for 3 days), and clonazepam (1 mg for 3 days).

**Outcomes:**

Remission of temporal lobe epilepsy symptoms and cognitive dysfunction was observed. Follow-up analysis of CSF and serological examination were not approved by the patient. His Mini-Mental State Examination score improved to 21/30. Stable remission of symptoms was achieved throughout the follow-up period of 50 days.

**Lessons:**

Autoimmune encephalitis (AE) should be considered in cases of late-onset epilepsy following meningioma peritumoral brain edema and resection. A diagnosis of AE should be considered in patients presenting with stroke-like symptoms if the magnetic resonance imaging abnormality does not match a known vascular territory. Early and correct diagnosis is crucial because immunotherapy is usually effective for this disease.

## 1 Introduction

Contactin-associated protein-like 2-antibody (CASPR2-Ab)-positive limbic encephalitis (LE) is a rare clinical disease capable of causing neuromuscular rigidity, Morvan syndrome, and LE and manifests as seizures, amnesia, cognitive disturbances, neuromyotonia, movement disorders, pain, and sleep disturbances.^[2]^ Peritumoral brain edema (PTBE) associated with meningiomas can lead to histological blood-brain barrier (BBB) breakdown. A rupture of the BBB in meningiomas may have a pathogenic effect by allowing antibodies to enter the central nervous system. Todd paralysis (a stroke-like presentation in some patients with epilepsy) caused by LE is not easily distinguished from acute ischemic stroke. Due to their broad clinical spectrum and variability in clinical imaging among patients, CASPR2-Ab-associated diseases may be underdiagnosed.^[3]^ By reporting this clinical course, we aim to emphasize that when meningioma patients still have epilepsy after meningioma resection, autoimmune encephalitis (AE) should be considered with an emphasis on identifying stroke mimics such as Todd paralysis caused by LE.

## 2 Case presentation

**Figure 1:**
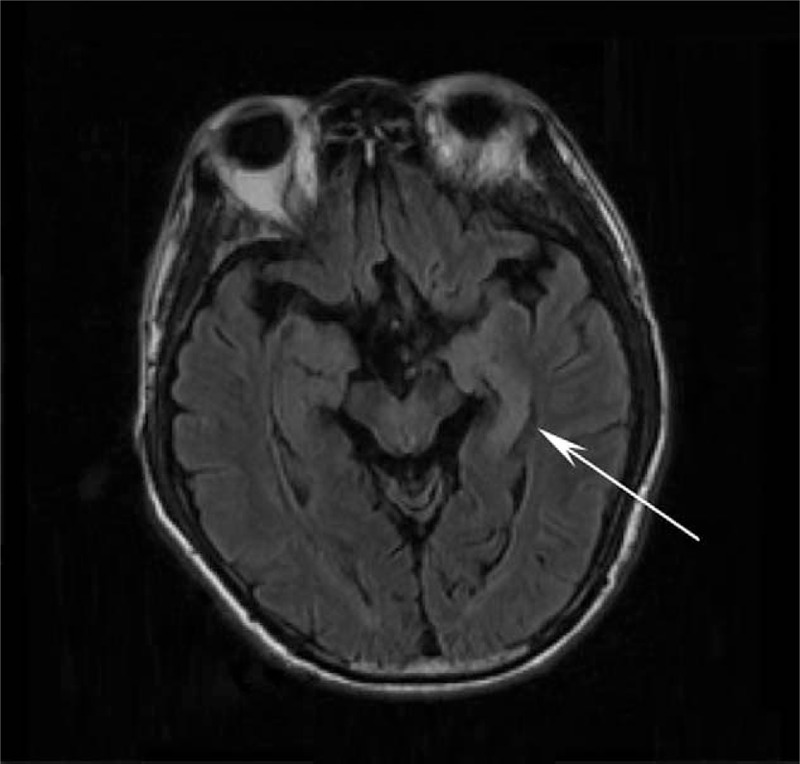
FLAIR. Cranial MRI images revealed left hyperintensity of the hippocampus on the FLAIR sequence. FLAIR = fluid-attenuated inversion recovery, MRI = magnetic resonance imaging.

**Figure 2:**
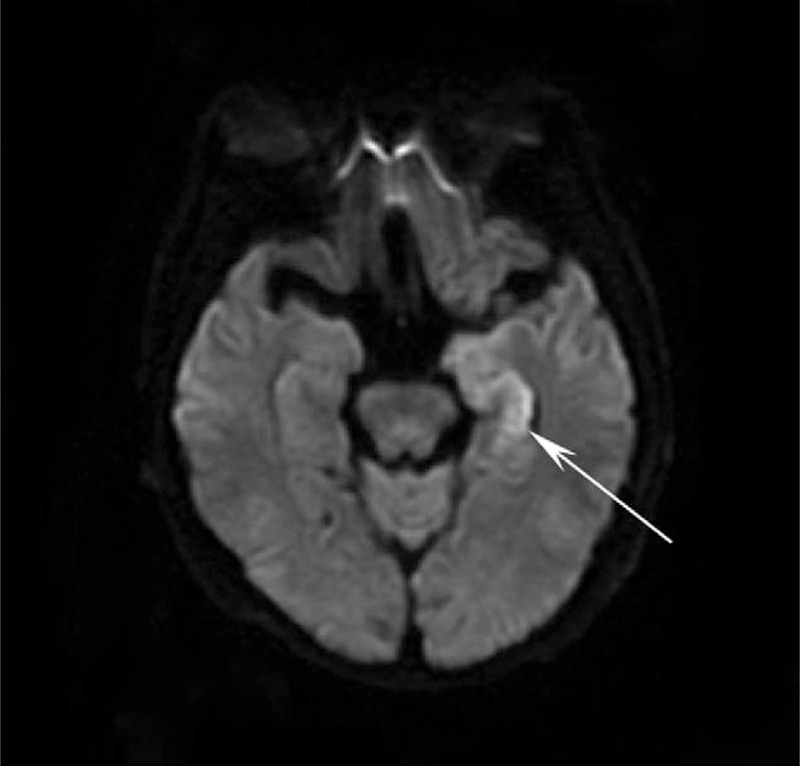
DWI. Cranial MRI images revealed left hyperintensity of the hippocampus on the DWI sequence. DWI = diffusion-weighted imaging, MRI = magnetic resonance imaging.

A 61-year-old man's past medical history was notable for 2 hospital admissions. He was admitted to a local emergency room on May 17, 2019, because of headaches, dysphoria due to aphasia, and mild postictal right-sided hemiparesis persisting for 20 minutes; after admission, he underwent a quick and complete recovery. Computerized tomography (CT) and magnetic resonance imaging (MRI) scans detected PTBE in the left temporal-parietal lobe. The patient underwent craniotomy for resection of the meningioma on May 21, 2019. The patient was readmitted to the local emergency room for right hemiparesis and aphasia for 2.5 hours on November 09, 2019. The patient exhibited occasional involuntary, repetitive, and paroxysmal contractions in the right limb. Three hours later, the patient had a generalized seizure, and anticonvulsive therapy was started. On diffusion-weighted imaging (DWI) and T2 fluid-attenuated inversion recovery (T2 FLAIR imaging), brain MRI demonstrated hyperintensity in the left hippocampus (Figs. 1 and 2). There were no vascular abnormalities according to cranial MRI. The patient's symptoms resolved, with the exception of cognitive deficits, on November 11, 2019, and the patient was discharged on November 14, 2019.

This patient presented to our clinic on November 23, 2019, with ictal-related increases in epigastric sensations, fear, and cognitive impairment, which had started 4 days before. The symptoms lasted from several minutes to several hours, and the attacks occurred frequently in a day. His Mini-Mental State Examination score was 16/30. We found deficits in multiple cognitive domains, including memory, computing, and executive functions. A cranial MRI FLAIR scan revealed mild abnormalities in the left hippocampus. Electroencephalography showed a few nonspecific slow waves in the background signals. Analyses of cerebrospinal fluid (CSF) for cells, glucose, protein, chloride, and culture were normal. Blood sodium was normal. Thyroid function and anti-thyroid antibodies, including anti-TG and anti-TPO, were negative. The tumor markers (CEA, AFP, CA125, CA19-9, CA15-3, SCCAg, NSE, Cyfra211, and TPS) and paraneoplastic neuronal antibodies (Hu, Ri, and Yo) were within the normal range, except that neuron-specific enolase levels were slightly higher than normal (16.04 ng/mL). An 18-fluorine fluorodeoxyglucose positron emission tomography scan showed hippocampal hypermetabolism. A body CT scan showed no malignancies. The patient's CSF samples had autoantibodies targeting contactin-associated protein-like 2 (CASPR2) at an immunoglobulin G titer of 1:320.

## 3 Discussion

Since the detection of autoantibodies against neuronal surface antigens, AE, a kind of encephalitis caused by specific neuronal antigens, has been more frequently diagnosed, especially in patients with symptoms of LE.^[4,5]^ Voltage-gated potassium channel (VGKC)-related antibodies are not directed at the VGKC itself but at 2 closely associated proteins, leucine-rich glioma-inactivated 1 (LGI1) and CASPR2.^[6]^

Todd paralysis (a stroke-like presentation in some patients with epilepsy) caused by LE is not easily distinguished from acute ischemic stroke. With typical temporal mesial hyperintensities, DWI of a stroke-like patient showed that there is a mismatch between diffusion restriction and the vascular territory, suggesting that it is a stroke mimic.^[1]^ An AE patient with VGKC antibodies after ischemic stroke has been reported, but in that case, the rupture of the BBB that occurs in stroke may have a pathogenic role by allowing antibodies to gain access to the central nervous system.^[7]^

In CASPR2 syndrome, tumors can occur at the same time, mainly related to thymoma, but more often no tumor is found. We describe, to the best of our knowledge, the first time in the literature a case of AE with CASPR2-Abs after meningioma PTBE and resection. Meningiomas are always accompanied by PTBE. To study PTBE, Piazza et al^[8]^ created a model that accurately simulates vasogenic brain edema and discovered that PTBE associated with brain tumors is predominantly a result of vascular endothelial growth factor (VEGF) secreted by brain tumors, and VEGF infusion can lead to histological BBB breakdown. Therefore, meningioma PTBE is intimately linked to BBB breakdown.

In a feline model of LE with antibodies against LGI1, a protein of the VGKC complex, Tröscher et al^[9]^ discovered that BBB leakage was present in limbic structures and those brain areas affected by BBB dysfunction revealed immunoglobulin and complement deposition as well as neuronal cell death. BBB breakdown may play a pathogenic role in AE. Here, meningioma PTBE inducing BBB dysfunction may have caused antigen exposure with subsequent immune response to multiple neuronal antigens and allowed antibodies to enter the central nervous system. Li et al^[10]^ reported that 13.5% of patients undergoing supratentorial meningioma surgery experienced postoperative seizures after discharge. Therefore, when meningioma patients continue to have late-onset epilepsy after meningioma resection, we should consider the possibility of AE. More clinical data are needed to support the abovementioned conjecture.

## 4 Conclusion

To the best of our knowledge, this is the first report of meningioma preceding CASPR2-Ab LE with a stroke mimic. In our patient, meningioma PTBE-induced BBB dysfunction may have been the cause of AE. When meningioma patients still have late-onset epilepsy after meningioma resection, the possibility of AE should be considered. Todd paralysis (a stroke-like presentation in some patients with epilepsy) caused by LE is not easily distinguished from acute ischemic stroke by clinicians in the emergency room.

## Author contributions

**Conceptualization:** Xiao-Jin Lu.

**Data curation:** Xiao-Jin Lu, Xian-Kai Xu.

**Formal analysis:** Yong-Xing Chen.

**Investigation:** Ran Li.

**Resources:** Xiao-Jin Lu, Yong-Xing Chen, Ran Li.

**Supervision:** Bao-Lin Shi.

**Writing – original draft:** Xiao-Jin Lu.

**Writing – review & editing:** Xian-Kai Xu.
